# Resilience of dermis resident macrophages to inflammatory challenges

**DOI:** 10.1038/s12276-024-01313-z

**Published:** 2024-10-01

**Authors:** Sang Hun Lee, David L. Sacks

**Affiliations:** Laboratory of Parasitic Diseases, National Institute of Allergy and Infectious Diseases, National Institutes of Health, Bethesda, MD USA

**Keywords:** Phagocytes, Mucosal immunology

## Abstract

The skin serves as a complex barrier organ populated by tissue-resident macrophages (TRMs), which play critical roles in defense, homeostasis, and tissue repair. This review examines the functions of dermis resident TRMs in different inflammatory settings, their embryonic origins, and their long-term self-renewal capabilities. We highlight the M2-like phenotype of dermal TRMs and their specialized functions in perivascular and perineuronal niches. Their interactions with type 2 immune cells, autocrine cytokines such as IL-10, and their phagocytic clearance of apoptotic cells have been explored as mechanisms for M2-like dermal TRM self-maintenance and function. In conclusion, we address the need to bridge murine models with human studies, with the possibility of targeting TRMs to promote skin immunity or restrain cutaneous pathology.

## Bridging murine models to humans: targeting TRMs for skin immunity

Our skin is more than just a physical shield; it’s a complex immune organ, filled with specialized cells like macrophages. In a detailed review, researchers explore these macrophages, focusing on their adaptability and function maintenance in the skin. This study synthesizes findings how macrophages interact with other immune cells, respond to inflammatory triggers, and contribute to tissue repair and homeostasis. The findings show that these macrophages can remain anti-inflammatory, even when faced with infections that usually trigger a strong immune response. They achieve this through various mechanisms, including interactions with specific immune cells that support their anti-inflammatory state, and engaging in processes that promote tissue repair without increasing inflammation. The researchers conclude that understanding these mechanisms opens new possibilities for treating skin diseases by targeting or mimicking the ways these macrophages control inflammation and support healing. This summary was initially drafted using artificial intelligence, then revised and fact-checked by the author.

## Introduction

The intricate landscape of cutaneous immunity is a testament to the complex interplay between various cell types that inhabit the skin, a barrier organ tasked with the unrelenting responsibility of protecting the body from external threats^1,2^. The foundational work of Metchnikov, who first characterized macrophages as phagocytes^3^, laid the groundwork for over a century of research into tissue-resident macrophages (TRMs) and their strategic positioning across barrier surfaces, including the skin. Recent discoveries have significantly enriched our understanding of macrophage functionality, demonstrating that these cells not only are passive scavengers engaged in host defense but are also actively involved in a range of homeostatic functions, including the resolution of inflammation and the promotion of tissue remodeling following damage. Moreover, it has been recognized that skin-resident macrophages primarily originate from embryonic precursors and maintain long-standing integration within the skin microenvironment^4,5^. This raises important questions about how they maintain their identity and function in the face of various inflammatory triggers, whether from infections, tumors, or mechanical or chemical stimuli. This review aims to shed light on the resilient adaptation of macrophages to their dynamic environment in the skin, exploring the mechanisms through which they crosstalk with other immune cells during various pathophysiological conditions.

## Main text

### Overview of skin architecture

The skin serves as the primary defensive barrier for all vertebrates and has undergone evolutionary specialization across different groups, such as aquatic vertebrates, reptiles, birds, and mammals^6^. The skin of vertebrates consists of three main layers, the epidermis, dermis, and hypodermis, which are populated by distinct populations of TRMs^7,8^. The epidermis is the surface layer, featuring a stratified squamous epithelium composed of keratinocytes bordered by a basal membrane. Langerhans cells are macrophages that reside in the epidermis and are seeded into the skin prenatally^9^. Unlike other TRMs, but similar to dendritic cells, they migrate to lymph nodes under homeostatic conditions and present antigens to antigen-specific T cells. The epidermis also houses melanocytes, Merkel cells, and nonepithelial immune cells, such as dendritic epidermal T cells. The underlying dermis is a connective tissue filled with fibroblasts and an extracellular matrix rich in collagen and elastic fibers that support the structural integrity, flexibility, and nourishment of the skin. Additionally, the dermis encompasses blood and lymphatic vessels, sebaceous and sweat glands, nerve endings, and hair follicles that extend from the epidermis. αβ and γδ T cells, mast cells, and ILC2s (innate lymphoid cells 2) are the major immune cells found in the dermis. Two main subsets of TRMs reside in the dermis: MR^high^ perivascular and Lyve1^high^ perineuronal macrophages^10–12^. Unlike Langerhans cells, these dermal macrophages remain stationary. Directly beneath the dermis lies a layer of fat cells known as dermal white adipose tissue. The deepest layer of the skin, the hypodermis or subcutaneous tissue, is divided into two distinct anatomical sublayers: the panniculus carnosus, which is a layer of skeletal muscle, and the interstitial connective tissue. Unlike the dermis, the hypodermis is primarily composed of CD11b^+^ myeloid cells, with hypodermal TRMs identified as the predominant cell type^13^.

### M2-like nature of dermal TRMs

The M1‒M2 paradigm, introduced by Mills et al. in 2000^14^, has often been misinterpreted as a rigid classification of macrophages rather than the “simplified operational concept” as it was intended^15^. Over time, our understanding has evolved into a spectrum model, where M1 and M2 macrophages represent two extremes, and most macrophages exist somewhere in between these extremes in vivo^16^. Within this spectrum, TRMs are generally classified as M2-like, reflecting their fundamental roles in the development, homeostasis, and resolution of inflammation^17^. This conceptualization is now supported by extensive experimental evidence, which includes profiling diverse subsets of TRMs alongside inflammatory monocyte-derived macrophages in the heart^18–20^, lung^21–23^, liver^24^, synovial tissue^25,26^, bladder^27^, spleen^28^ and adipose tissue^29,30^. Dermal TRMs, in particular, exhibit anti-inflammatory properties, such as inefficient antigen presentation to T cells, a lack of proinflammatory mediator production in response to inflammatory stimuli, and an inability to control intracellular pathogens, for which they can provide a replicative niche^10,11,31–33^. Thus, most TRMs preserve their M2-like characteristics amid diverse inflammatory challenges, in contrast with the widely recognized general plasticity of macrophages.

### Ontogeny of dermal TRMs

For many years, the prevailing belief was that macrophages represent a fairly uniform group of mononuclear phagocytes originating from hematopoietic stem cells (HSCs) in the bone marrow^34^. Their main role is to safeguard organs against infections. However, studies in the 1980s began to challenge this view, revealing that tissue macrophages are not terminally differentiated^35–37^. It is now recognized that some macrophages are capable of proliferating, allowing for long-term self-renewal in situ without contribution by monocytes. This shift in understanding began with human Langerhans cells, which were discovered to be of donor origin years after a hand transplant^38^, a finding later confirmed in murine Langerhans cells^39,40^. Subsequent research revealed similar in vivo proliferation capabilities in TRMs located in the dermis^11^, brain^41,42^, peritoneum^43,44^, lung^45^, spleen^45^, adipose tissue^46^, and heart^47^. The introduction of innovative fate-mapping mouse models has revolutionized our understanding of macrophage biology over the past decade. When paired with systems biology approaches, these models enable the longitudinal tracking of macrophages from their progenitor stages to their mature states within their specific organs of residence^48,49^, indicating prenatal seeding and local maintenance of TRMs in peripheral tissues. In mice, embryonic development day 8.5 (E8.5) marks the beginning of a process in which yolk sac erythromyeloid progenitors (EMPs) generate premacrophages (pMacs)^50,51^. These pMacs then colonize embryonic tissues from E9.0 onward and further differentiate into tissue-specific macrophages during organogenesis. EMPs are also responsible for the production of fetal monocytes, which in turn contribute to the formation of tissue-specific macrophage populations^52^. Notably, most TRMs undergo local proliferation and are characterized by their long lifespan during the steady state of adulthood. The gastrointestinal tract presents an exception, with its primary TRMs originating from blood monocytes in a steady state^53^. Following severe inflammation, embryonic-derived TRMs may be supplanted by monocyte-derived cells that adopt similar markers and transcriptional profiles to TRMs^54,55^. The dynamic transition from monocytes to TRMs was recently delineated in vivo through time-course single-cell RNA sequencing (scRNA-seq)^56^.

Dermal TRMs exhibit a distinct developmental pattern within specific tissue compartments, such as those associated with blood vessels or sensory nerves. Two major subsets of macrophages have been identified in the adult dermis, each with unique functions and locations on the basis of surface marker expression and transcriptional profiles^10–12,31,57^: (1) vessel-associated TRMs, which are MHCII^low^CX3CR1^low^LYVE1^high^MR^high^, and (2) sensory-nerve-associated TRMs, which are MHCII^high^CX3CR1^high^LYVE1^low^MR^low^. Although E8.5 fetal macrophage progenitors initially populate the dermis, studies suggest that postnatal dermal macrophages are partially replenished by monocyte-derived macrophages. The turnover rates of these cells during adulthood vary by compartment, with MHCII^high^CX3CR1^high^LYVE1^low^MR^low^ sensory-nerve-associated TRMs seeded prenatally but then continuously replaced by blood-circulating monocytes. In contrast, recruitment of the MHCII^low^CX3CR1^low^LYVE1^high^MR^high^ perivascular population occurs mainly during embryogenesis, with minimal replacement during adulthood.

### Perivascular dermal TRMs

Each macrophage population performs specific roles tailored to its subtissue niche and the demands of its tissue environment. In multiple tissues, perivascular TRMs often have important functions related to their perivascular position, such as the scavenging of blood-borne self and foreign materials, the regulation of vascular permeability, and the control of the movement of other leukocytes across the vasculature^31,58^. We demonstrated the highly phagocytic nature of MR^high^ dermal TRMs, which rapidly scavenged high-molecular-mass dextran from the blood lumen^10^. This activity was reported to be mediated by transendothelial protrusion^31^. Some MR^high^ dermal TRMs acquire *Leishmania major* infections through transfer from or efferocytosis of parasitized neutrophils, which swarm into sand fly transmission sites in the skin^59^. This phenomenon offers direct evidence for the “Trojan horse” model of *Leishmania* infection, which involves the immunologically silent infection of macrophages *via* receptors engaged in the capture of apoptotic cells^60^. Dermal TRMs are also the only cells capable of capturing and retaining tattoo pigment particles for long-term tattoo persistence, further indicating their high phagocytic activity without inducing local inflammation^61^.

Pericytes, which surround the endothelial cells of small blood vessels, have long been recognized for their role in regulating vascular permeability and facilitating neutrophil movement during their exit from vessel lumens^62,63^. Perivascular TRMs are also emerging as significant contributors to vascular homeostasis. Accordingly, multiple scRNA-seq and bulk RNA-seq analyses have indicated that perivascular TRMs express relatively high levels of genes linked with alternative activation, wound healing, repair, and fibrosis in addition to high bystander IL-10 secretion^12,33^. In experiments with clodronate liposome-treated mice or genetically engineered mice lacking perivascular TRMs, vascular instability, and increased permeability were observed, a phenotype reversible *via* the adoptive transfer of TRMs^64,65^. These cells are instrumental in promoting eosinophil migration into the dermis infected by *L. major*, primarily through the production of CCL24, also known as eotaxin-2^32^. Notably, perivascular dermal TRMs appear to facilitate neutrophil migration into skin infected with *Staphylococcus aureus*^66^. Neutrophils extravasate from inflamed dermal venules near perivascular macrophages, which are significant sources of the neutrophil chemoattractants *Cxcl1*, *Cxcl2*, *Ccl2*, *Ccl3*, and *Ccl4*. While dermal TRMs do not produce IL-1β in this setting and other aspects of their M2-like functionality may be retained, these findings suggest that the strategic perivascular positioning of these TRMs may facilitate proinflammatory leukocyte infiltration.

### Perineuronal dermal TRMs

Historically, the identification of macrophages within peripheral nerves, such as the sciatic nerve and dorsal root ganglion (DRG), has relied on the assessment of classic macrophage markers, including CR3 and MHC-II^67,68^. Recent studies have provided a more comprehensive characterization of TRMs associated with perineuronal environments^69,70^. Notably, CX3CR1^GFP/+^ perineuronal TRMs have been observed across various neuronal compartments, including the sciatic nerve, DRG, and cutaneous intercostal fascial nerves. Transcriptome analyses further revealed that perineuronal TRMs share certain features with activated microglia while also expressing unique genes associated with angiogenesis, collagen fibril organization, peripheral nerve structure, and axon guidance^69^. These expression signatures suggest a role for perineuronal, dermal TRMs in facilitating axon sprouting following cutaneous nerve injury by breaking down myelin from damaged fibers^70^. Axons regrowing at injury sites attract macrophages from various dermal locations, which then gradually adopt a sensory-nerve-associated phenotype.

Recent discoveries further emphasize the interaction between peripheral neurons and perineuronal dermal TRMs in both pain sensation and macrophage-mediated tissue repair. Nociceptor endings extend into injured skin and muscle tissues, signaling to immune cells through the neuropeptide calcitonin gene-related peptide (CGRP) during the healing process^71^. CGRP acts *via* receptor activity-modifying protein 1 (RAMP1) on macrophages to polarize them toward a prorepair phenotype. Additionally, GINIP^+^ sensory neurons secrete TAFA4, a neuropeptide that promotes dermal TRM anti-inflammatory functions^72^. Moreover, dermal TRMs influence nociception thresholds through the regulation of neurotrophic nerve growth factor (NGF) levels^73^. Future investigations should prioritize unraveling the intricacies of the molecular interactions between perineuronal TRMs and peripheral neurons, particularly within the framework of skin infections. For example, in the context of *S. aureus* skin infection, TRPV1^+^ nociceptors have been shown to suppress neutrophil recruitment and modulate skin macrophage polarization toward the M2 phenotype through the release of CGRP^74^.

### Self-maintenance of M2-like dermal TRMs

**Fig. 1:**
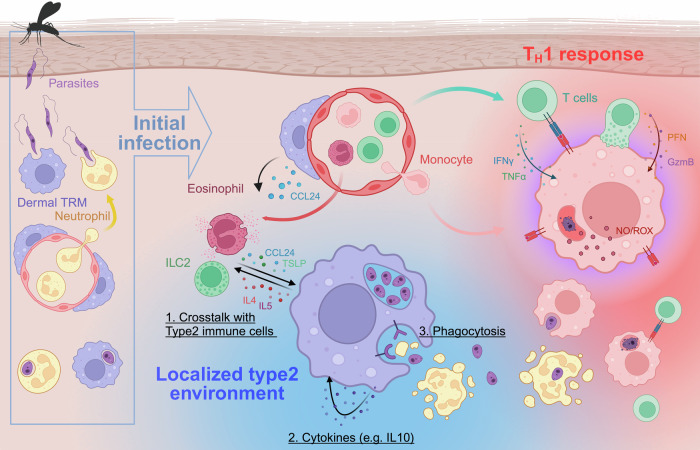
The mechanisms by which dermal TRMs maintain an M2-like phenotype and serve as a replicative niche for *L. major.* The left side of the figure illustrates the initial establishment of *L. major* infection within the dermis. The transmitted metacyclic form of *L. major*, introduced by either an infected sand fly or experimental needle injection, is quickly phagocytosed by TRMs or by neutrophils that swarm to the transmission site. Inside the phagolysosome, the parasite transforms into the amastigote form, which is the intracellular stage of *Leishmania* in the mammalian host. Inflammatory monocytes are subsequently recruited to the site and become the dominant cells harboring parasites during the development of the cutaneous lesion. Following a robust T_H_1 immune response induced in the local draining lymph nodes, antigen-specific CD4^+^ and CD8^+^ T cells are recruited back to the site and release mediators, including IFNγ, TNFα, granzyme B (GzmB), and perforin (PFN), which activate infected monocytes and monocyte-derived cells to kill the parasite *via* their production of reactive oxygen species (ROS) and nitric oxide (NO). In contrast, TRMs employ various mechanisms to maintain their M2-like properties and promote parasite persistence, including their capacity to (1) orchestrate localized interactions with type 2 immune cells, including group 2 innate lymphoid cells (ILC2s) and eosinophils; (2) produce anti-inflammatory cytokines (e.g., IL-10) that operate in an autocrine fashion; and (3) engage in apoptotic cell clearance *via* receptors, including the TYRO3/AXL/MERTK family of receptor tyrosine kinases, which imprint an anti-inflammatory program on TRMs.

It has been suggested that the factors influencing TRM identity can be classified into two main categories: (1) intrinsic factors, which include the ontogeny of adult TRMs consisting of a specific combination of cells derived from both embryonic precursors and adult monocytes; and (2) extrinsic local factors, which are unique to the TRM niche of residence, including the presence of local inflammation^5^. When considering local factors, recent findings suggest that TRMs are not merely passive recipients of signals from their local microenvironment. Rather, they also actively interact with their immunological niche, shaping their own epigenetics for self-maintenance. This finding indicates a more dynamic role whereby TRMs influence and adapt to their surroundings, contributing to the resilience and specificity of local immune responses. We propose that the self-maintenance of M2-like dermal TRMs involves three key mechanisms: (1) interactions with type 2 immune cells, (2) cytokine-mediated autocrine regulation, and (3) self-imprinting through phagocytosis. This conceptual framework effectively explains how localized anti-inflammatory environments are orchestrated by perivascular TRMs. The significance of these mechanisms is highlighted by the ability of M2-like dermal TRMs to provide a replicative niche for intracellular pathogens, even in the presence of strong type 1 inflammation. Further details of these actions are discussed below and are graphically summarized in Fig. 1.

### Crosstalk with local type 2 immune cells

While the role of IL-4 produced by innate cells has been mostly studied in the context of T_H_2 cell differentiation^75,76^, the effects of the innate sources of IL-4 on macrophages have also been described under both homeostatic and inflammatory conditions. A pivotal discovery by Jenkins et al. revealed that IL-4 directly triggers the self-proliferation of TRMs in the context of T_H_2-related pathologies^44^. Basophils reportedly provide the IL-4 required for the local proliferation of monocyte-derived macrophages that replace Kupffer cells lost during bacterial infection^24^. In the skin, Ly6C^+^CCR2^+^ inflammatory monocytes have been shown to differentiate into M2-like macrophages under the influence of basophil-derived IL-4 after helminth infection or allergen challenge^77,78^. Eosinophils were also found to play an important role in maintaining alternatively activated macrophages in adipose tissue through an IL-4-dependent process^30^. ILC2s in peripheral tissue are important regulators of eosinophil survival and recruitment because of their constitutive expression of IL-5 and its upregulation during type 2 inflammation^79^. Accordingly, ILC2-derived IL-5 is implicated in the homeostatic homing of eosinophils into the small intestine and visceral adipose tissue and, subsequently, in sustaining alternatively activated tissue macrophages implicated in metabolism^29^.

We identified eosinophils and ILC2s as active secretors of IL-4 and IL-5, respectively, and the cytokines from these cellular sources are critical for maintaining MR^high^ M2-like, dermal TRMs as critical host cells for the growth and persistence of *L. major* during cutaneous infection^32^. Critically, dermal TRMs actively orchestrate the activities of eosinophils and ILC2s in infected skin. The close physical association between eosinophils and dermal TRMs is facilitated by CCL24 (eotaxin-2), a chemokine with potent chemotactic activity for eosinophils^32^. CCL24 was first identified in an mRNA library from activated monocytes^80,81^. Our findings demonstrate that, among various innate and adaptive immune cell populations, as well as nonhematopoietic cells, dermal TRMs are the sole producers of CCL24 in the skin. Our studies also revealed that dermal TRMs produce thymic stromal lymphopoietin (TSLP), one of the three key alarmins previously recognized as strong activators of ILC2s (along with IL-25 and IL-33), which is generally thought to be produced by epithelial and stromal cells in response to insults at barrier surfaces^33,82^. Neutralizing TSLP with antibodies or genetically removing TSLP specifically in dermal TRMs leads to a decrease in both the number of ILC2s and their interactions with dermal TRMs in infected skin, ultimately reducing the number of dermal TRMs and promoting greater control of the infection. Given that ILC2s reside within the perivascular niche in the dermis^33^, this proximity may be encouraged by direct TSLP release from perivascular TRMs. This colocalization echoes previous findings where the accumulation and function of ILC2s around lung bronchi and larger vessels were attributed to IL-33 and TSLP production by adventitious stromal cells (ASCs)^83^. The possible redundancy of TSLP from perivascular TRMs and ASCs in enhancing ILC2 activity remains an open question. Other TRMs have been identified as sources of infection-driven alarmins, such as IL-33 produced by alveolar macrophages in response to respiratory viruses, further challenging the notion that alarmin production is exclusive to epithelial cells^84,85^. Thus, TRMs seem to play a crucial role in the production of alarmins to activate ILC2s in tissues that are more anatomically distant from epithelial barriers, such as the dermis or lung parenchyma.

### Autocrine control of cytokines

Cytokines play crucial roles in modulating many macrophage functions, including those related to inflammation and tissue repair. Importantly, macrophages can be a source of cytokines themselves. Studies have highlighted TRMs as key regulatory cells that spontaneously secrete IL-10, which is expressed in both perivascular and perineuronal TRMs across multiple tissues, including the dermis^11,12^. The autocrine IL-10 pathway in TRMs has been well characterized for its anti-inflammatory properties. For example, peritoneal and intestinal TRMs actively suppress the release of the proinflammatory cytokines IL-1β, IL-12, and IL-23 through this pathway^86–88^. We also showed that IL-10 signaling is crucial for maintaining M2-like dermal TRMs, although the autocrine effects of IL-10 are not yet fully understood^10^. Both dermal TRMs and T cells are significant sources of IL-10 in the *L. major*-infected dermis. TGF-β, another self-regulatory cytokine, promotes the differentiation, maturation, and self-maintenance of alveolar, epidermal, and colonic TRMs^89–91^. Autocrine TGF-β receptor signaling leads to the upregulation of PPAR-γ, a key transcription factor essential for the development of alveolar macrophages. Additionally, autocrine IL-15 has been shown to suppress proinflammatory cytokine production from peritoneal TRMs^92^.

### Phagocytic activity

TRMs are renowned for their potent phagocytic capabilities, including rapid and immunologically silent clearance of apoptotic cells (ACs)^93^. AC sensing and clearance, which can stimulate the production of IL-10 by macrophages^94^, are critical for maintaining tissue homeostasis and preventing inflammatory responses that could lead to autoimmunity. TRMs exhibit characteristics conducive to this process, such as high expression of AC recognition receptors, low expression of Toll-like receptor 9 (TLR9), and diminished responsiveness to nucleic acids^95^. These features are transcriptionally governed by Kruppel-like factors 2 (KLF2) and 4 (KLF4), which regulate the expression of genes involved in the recognition and clearance of ACs. Importantly, the phagocytosis of unwanted cells aids in the maintenance of tissue homeostasis and also plays a crucial role in imprinting an anti-inflammatory profile on TRMs^96^. Apoptotic clearance across several tissues is impaired in the absence of the nuclear receptors LXRα, LXRβ, and PPARγ, which increase the phagocytic capacity of macrophages and are crucial for preventing excessive inflammation and autoimmunity^97,98^. Furthermore, after wounding caused by migrating helminths in the lung, the clearance of apoptotic cells by the TYRO3/AXL/MERTK family of receptor tyrosine kinases, combined with IL-4/13 signaling, initiates anti-inflammatory and tissue repair responses in alveolar macrophages^99^. Similarly, we showed that the anti-inflammatory programming of dermal TRMs is supported by AXL/MERTK-dependent engulfment of apoptotic neutrophils, along with IL-4/IL-13 signaling^32,59^.

### Murine models to study dermal TRMs

Functional studies on TRMs in murine models can require specific ablation of these cells in a manner that is selective for resident populations vs. monocyte-derived cells and that may also be tissue-specific. Macrophage depletion *via* clodronate-loaded liposomes is a common method for exploring the in vivo functions of macrophages in mice^100,101^. With clodronate, macrophages undergo apoptosis after phagocytosing clodronate-containing liposomes, typically resulting in substantial macrophage depletion within 24–48 h after a single injection. However, clodronate-loaded liposomes can also be taken up by other phagocytic cells, such as circulating monocytes and dendritic cells, leading to potential off-target effects^102,103^. Neutrophils can also take up clodronate-loaded liposomes; although they are not depleted, their function is significantly impaired^104^. Systemic antibody treatment that blocks CSF-1/CSF-1R interactions is another commonly used method to ablate TRMs, as colony-stimulating factor-1 (CSF-1, also known as macrophage-CSF) is a crucial regulator of the survival, proliferation, differentiation, and function of mononuclear phagocytes^105^. Several strategies are employed to inhibit CSF-1 activity, including the use of inhibitors that target the protein tyrosine kinase activity of the receptor, as well as antibodies directed against the receptor or the ligand itself^106^. However, caution is again needed with this approach since blocking CSF-1/CSF-1R interactions can have adverse effects on many aspects of monocyte biology, such as differentiation and proliferation, in addition to its role in depleting embryonically derived TRMs.

Achieving cell-type specificity in genetic deletion models can also be challenging. Cre transgenic models driven by a single promoter often lack specificity for their intended target cells. This limitation is particularly apparent in the study of myeloid immune cells given the diverse and closely related subtypes of TRMs^107^. Sequential double promoter systems, such as MM^DTR^ (*Lysm*^*Cre*^ *x Csf1r*^*LsL-DTR*^) and *Cx3cr1*^*CreER/+*^*x Csf1r*^*Flox/Flox*^, effectively delineate resident macrophages across multiple tissues but also include monocytes and other myeloid cells^108,109^. A binary system using *Lyve1*^*ncre*^ with *Cx*_*3*_*cr1*^*Cre*^ targets perivascular TRMs in the brain and other tissues, but this approach suffers from low efficiency, achieving only 5% expression penetrance in the targeted TRMs^110^. Although the discovery of TRM-specific genes has facilitated the development of new Cre transgenic models that enable the selective visualization or depletion of TRMs in the liver or brain^111,112^, a Cre transgenic system specifically targeting dermal TRMs has not yet been established. Recently, we developed a single-promoter-driven Cre system using the *Ccl24* promoter to specifically visualize dermal TRMs in both steady and infectious states^33^. Flow cytometric analysis of *Ccl24-Cre*: *ROSA26-LSL-tdTomato* mice during *L. major* infection revealed that approximately 80% of MHCII^low^MR^high^ dermal TRMs were tdTomato^+^, whereas other skin myeloid populations were not labeled.

While *Ccl24* expression appears to be confined to MR^high^ resident macrophages in the skin, its expression is not tissue-specific. Under naïve conditions, *Ccl24-Cre* mice also allow for the visualization of TRMs from other tissues. The labeled TRMs were predominantly found in two distinct subtissue niches in multiple tissues: perivascular areas, including various organ-specific TRMs, and tissue linings, such as serosal membranes of the lung and visceral adipose tissue. The expression of *Ccl24* by perivascular and serosal TRMs across tissues in physiological homeostasis likely indicates a shared aspect of their alternative activation states. For example, serosal TRMs in the synovial cavity and peritoneal serosa play a tissue-protective role by sequestering inflammatory responses against local inflammation or tissue damage^26,113^. The constitutive expression of *Ccl24* by TRMs across different tissues suggests a shared role for eosinophil interactions in maintaining their M2-like phenotypes even under steady-state conditions.

### Dermal TRMs in humans

Understanding the structural differences between mouse and human skin is crucial for interpreting cross-species research findings. Mouse skin is characterized by a dense distribution of hair follicles within its epithelium, which contrasts with the predominant interfollicular epithelium of human skin^114^. The mouse epidermis is thinner, typically consisting of 2–3 layers of keratinocytes, and is only approximately a quarter of the thickness of the human epidermis^115,116^. While the epidermal layers in both species contain dendritic, antigen-presenting Langerhans cells, along with CD4^+^ and CD8^+^ T cells, a unique characteristic of mice is the presence of dendritic epidermal T cells (DETCs) that have monoclonal Vγ3Vδ1 T cell receptors^117^. These differences also extend to nonepithelial skin components. The human dermis is significantly thicker than that of mice, has fewer hair follicles, and exhibits distinct healing properties; notably, mouse skin can regenerate effectively with minimal scarring^7^. Advances in techniques such as scRNA-seq and spatial transcriptomics have highlighted variances in subpopulation composition and intercellular communication between mouse and human skin^118^. The impact of these structural differences on the skin immune response and their implications for immune system function continues to be the subject of active research.

Using scRNA-seq of human fetal myeloid cells, Bian et al. identified two developmental trajectories: yolk sac-derived primitive macrophages that migrate early to the head region as microglial precursors and yolk sac-derived myeloid-biased progenitors (YSMPs) that develop within the fetal liver^119^. This latter group gives rise to granulocyte‒monocyte progenitors (GMPs), myeloblasts, and subsequently fetal liver monocytes that initiate tissue residency programs. The expression of specific genes, such as SPIC in liver macrophages, CCL13 in blood macrophages, and the tissue-related genes BMX in the skin and MMP1 in lung macrophages, further delineates their specialization. scRNA-seq profiling of human fetal skin has revealed similar hematopoietic waves: primitive macrophages from yolk sac-derived progenitors and tissue definitive macrophages from fetal liver monocytes^120^. These data suggest that early embryonic macrophage development in humans closely mirrors that observed in mice. Studies involving human organ transplants have provided valuable insights into the longevity and origin of tissue macrophages. For example, donor-derived alveolar macrophages have been identified in lung transplant recipients two years posttransplantation, with a fraction of these cells proliferating locally alongside recipient-derived monocytes that acquire macrophage properties^121^. In liver transplants, donor-derived macrophages can persist for up to ten years and express higher levels of CD206, CD163, and Cx_3_CR1 than can recipient-derived liver macrophages, which originate from infiltrating monocytes and exhibit higher levels of CCR2, indicating their hematopoietic origin^122^. The human heart also features distinct macrophage populations differentiated by CCR2 expression; CCR2^−^ macrophages are tissue-resident, capable of lifelong self-renewal through local proliferation, and are involved in reparative functions, whereas CCR2^+^ macrophages are maintained through the recruitment of monocytes and are associated with inflammatory responses^18^.

CD163^high^HLA-DR^low^ was first identified as a surface marker for dermal TRMs in human skin, exhibiting characteristics similar to those of their murine counterparts^123^. These include a lack of capacity to stimulate T cell proliferation and the distinct accumulation of granular pigments, which are often noted in biopsies from tattooed skin^11,61^. In terms of ontogeny, CD163^+^ dermal TRMs from graft-vs.-host skin lesions after allogeneic hematopoietic stem cell transplantation presented a mixed composition of donor vs. host origins, both of which strongly expressed tissue-remodeling genes, such as *IL-10* and *VEGFA*^124^. Interestingly, preexisting progenitors, such as CD34^+^ cells, have been identified in human skin and can generate dermal TRMs de novo under conditions of neurogenic inflammation rather than from self-proliferating or extravasating monocytes, as observed in a mouse model^125^. Recently, further complexity has increased our understanding of human dermal TRMs through the identification of multiple subsets of dermal macrophages. On the basis of scRNA-seq analysis of human skin biopsies associated with chronic inflammatory diseases, such as psoriasis and atopic dermatitis^126–129^, dermal TRMs can be subclustered into 2–4 distinct groups depending on the study. In addition to CD14^+^ monocyte-derived macrophages^130^, these cells are largely divided into a population expressing complement receptors and scavenger receptors, such as CD163, and another population characterized by the expression of transcription factors associated with alternative activation, such as *NR4A1*, *NR4A2*, and *KLF4*, with transcriptional profiles more similar to those of fetal skin macrophages. Our extended analysis of one of the human scRNA-seq datasets^126^ provided evidence that cells in the macrophage clusters marked by *Mrc1* expression also coexpressed *Tslp* and *Ccl24* in both healthy and allergic skin biopsies^33^. This finding aligns with our mouse data and with the detection of high levels of *Tslp* expression in perivascular CD163^+^ macrophages from patients with diffuse cutaneous systemic sclerosis^131^. Further research is necessary to associate each subset of human dermal TRMs with tissue-specific homeostatic and immunological functions and to explore how their development may vary throughout life or in response to infections or inflammatory signals.

### Concluding remarks

Our skin is vital to our health, functioning as an immune organ that defends against relentless external threats. Dermal TRMs exemplify both the responsive and homeostatic nature of the skin immune system, are prepositioned to phagocytose noxious substances and microbes and are also programmed to regulate inflammation and tissue repair. This review highlights the self-regulating functions of dermal TRMs and the localized immune circuitries that help maintain their anti-inflammatory program, which can operate even in strongly proinflammatory environments, such as *L. major*-infected skin. While these functions are particularly critical to the skin as an external barrier, they are shared with TRMs in the intestines, lungs, and other mucosal barriers. There is an urgent need to translate findings from mouse models to human skin, refine the markers used to identify dermal TRMs, and employ tools such as single-cell and spatial transcriptomics to better define the function of these cells in pathological settings. A deeper understanding of these mechanisms will improve our ability to therapeutically modulate dermal TRMs, their interacting cells, and mediators, either to promote antimicrobial immunity or to restrain cutaneous pathologies.
